# Efficiency and safety of hepatic arterial infusion chemotherapy (HAIC) combined with anti‐PD1 therapy versus HAIC monotherapy for advanced hepatocellular carcinoma: A multicenter propensity score matching analysis

**DOI:** 10.1002/cam4.6836

**Published:** 2024-01-09

**Authors:** Yangyang Li, Wendao Liu, Junwei Chen, Yongxin Chen, Jiandong Guo, Huajin Pang, Wentao Zhang, Chao An, Chengzhi Li

**Affiliations:** ^1^ Department of Interventional Radiology and Vascular Surgery The First Affiliated Hospital of Jinan University Guangzhou Guangdong P. R. China; ^2^ Department of Interventional Therapy Guangdong Provincial Hospital of Chinese Medicine and Guangdong Provincial Academy of Chinese Medical Sciences Guangzhou Guangdong P. R. China; ^3^ Department of Interventional Radiology The Third Affiliated Hospital of Sun Yat‐Sen University Guangzhou Guangdong P. R. China; ^4^ Division of Vascular and Interventional Radiology, Department of General Surgery Nanfang Hospital, Southern Medical University Guangzhou Guangdong P. R. China; ^5^ Department of Radiology The First Affiliated Hospital, Nanchang University Nanchang Jiangxi P. R. China; ^6^ Department of Minimal Invasive Intervention Sun Yat‐sen University Cancer Center; State Key Laboratory of Oncology in South China; Collaborative Innovation Center for Cancer Medicine Guangzhou P.R. China

**Keywords:** hepatic arterial infusion chemotherapy, hepatocellular carcinoma, immune checkpoint inhibitors, propensity score

## Abstract

**Purpose:**

To investigate the clinical efficacy and safety of combination therapy of hepatic arterial infusion chemotherapy (HAIC) and anti‐programmed cell death protein‐1 (PD‐1) therapy in the treatment of advanced hepatocellular carcinoma (HCC).

**Methods:**

In this retrospective clinical research, from March 2018 to December 2019, 1158 HCC patients categorized as BCLC stage C were reviewed for eligibility. We utilized propensity score matching (PSM) to mitigate initial disparities between the groups. The evaluation of the best tumor response was conducted in accordance with mRECIST 1.1 criteria. The difference in survival outcomes including overall survival (OS), progression‐free survival (PFS), and objective response rate (ORR) between groups were compared.

**Results:**

Following the eligibility review, 453 patients underwent a combined treatment of HAIC with PD1 inhibitors (HAIC‐PD1 group), while 221 patients received HAIC monotherapy (HAIC group) met the inclusion criteria and were finally enrolled in this study. In the entire cohort, the HAIC‐PD1 group exhibited significantly prolonged overall survival (median overall survival: 40.4 months vs. 9.7 months, *p* < 0.001) and progression‐free survival (median progression‐free survival: 22.1 months vs. 5.8 months, *p* < 0.001). By propensity score, patients were matched according to baseline differences, resulting in all 442 patients in group HAIC‐PD1 (*n* = 221) and group HAIC (*n* = 221). After PSM adjustment, as well, the survival of the HAIC‐PD1 group was still distinctly longer than the HAIC group (median overall survival time, 40.4 months vs 9.7 months, *p* < 0.001; median progression‐free survival, 22.1 months vs 5.7 months, *p* < 0.001). Univariate and multivariable analysis demonstrated that AFP level, metastasis, and therapeutic schedule were independent predictive factors for overall survival.

**Conclusion:**

The combination therapy of HAIC and PD1 inhibitors successfully extended OS to advanced HCC patients and could be a better choice than HAIC monotherapy.

## 1 INTRODUCTION

Hepatocellular carcinoma (HCC), the prevailing histological variant of liver malignancy, ranked as the sixth neoplasm incidence, is identified as the third leading mortality attributable to cancer worldwide.^1^, ^2^ From an epidemiological standpoint, there are many risk factors for primary liver malignancy, among which hepatitis B virus infection and alcoholic hepatitis are the main factors in Asia and Western countries respectively.^3^, ^4^ Unfortunately, due to the highly insidious nature of HCC, a majority of cases have progressed to an advanced stage upon diagnosis and precluded the opportunity for curative local intervention.

Among limited treatment options available, neither monotherapy with recommended first‐line multi‐kinase inhibitors such as lenvatinib and sorafenib, nor with second‐line immune checkpoint inhibitors mainly represented by nivolumab attained favorable outcomes.^5^, ^6^, ^7^, ^8^ Moreover, traditional chemotherapy has a wide range of actions, unstable efficacy, serious systemic adverse reactions, and limited selectivity of individualized treatment options, which make it infrequently enrolled in medical practice. Formerly, several phase III trials of individual chemotherapy all failed to express a positive outcome.^9^, ^10^, ^11^ In this setting, hepatic artery infusion chemotherapy (HAIC) is regarded as a potential substitute chemotherapy strategy. During the HAIC procedure, chemotherapeutic agents are administered directly into the tumor blood supply artery trunk through the pre‐implanted microcatheter within the liver, so as to avoid the first‐pass effect and constitute a superior systemic chemotherapy characteristic with elevated regional drug concentration and minimal systemic adverse effects. Despite undiminished international controversy, HAIC has been endorsed as a viable therapeutic regimen for advanced HCC by the Japan Society of Hepatology (JSH).^12^ It is noteworthy that advanced HCC patients are more commonly present with poor hepatic function. In this patient population, HAIC appears to demonstrate sufficient tolerance and promising therapeutic effects.^13^ In a phase III randomized trial, the HAIC‐FOLFOX regimen demonstrated a median overall survival (OS) of 13.9 months, which surpassed the median OS of 8.0 months observed in the sorafenib group. The implementation of this treatment approach realized superior management of advanced HCC.^14^ In recent times, the emergence of immunotherapy has garnered significant attention as a promising therapeutic modality for various solid tumors. For chronic inflammation‐induced immune surveillance and dysregulation of the immune environment, programmed death‐1 (PD‐1) inhibitors can restrain tumor immune evasion by blocking the PD‐1/PD‐L1 pathway, increasing the intracorporal anti‐tumor immunoreaction.^15^ In spite of the monotherapy response rate of less than 30% in HCC,^16^ immunotherapies yielded remarkable prospects when combined with other treatment modalities, and the capability of HAIC in enhancing the effectiveness of targeted‐ and immune‐therapy has already been confirmed.^17^, ^18^, ^19^ To date, the combined immunotherapeutic approach for HCC remains in the investigational phase and necessitates additional monitoring and scrutiny.

Here, we undertook such a retrospective study to expand upon the existing evidence base regarding the efficacy of combined HAIC and anti‐PD1 treatment for advanced HCC patients. Specifically, we sought to examine the potential prognostic benefits of this therapeutic approach across various subgroups of HCC patients, with the ultimate goal of furnishing clinicians with an objective reference point for clinical decision‐making.

## 2 METHODS

### 2.1 Clinical information

The Institutional Review Committees at each study center provided ethical approval for this retrospective multicenter study. The present investigation adhered to the principles outlined in the Declaration of Helsinki.

The eligibility of 1158 patients diagnosed with advanced HCC, who underwent treatment with either a combination of HAIC and PD1 inhibitors or HAIC monotherapy, was evaluated through the review of clinical data from four hospitals between March 2018 and December 2019.

The inclusion criteria for this study encompassed the following parameters: (1) diagnosis of HCC confirmed through histopathological biopsy or precise radiological findings in line with the American Association for the Study of Liver Disease (AASLD) guidelines.^20^, ^21^ (2) Age ranging from 18 to 80 years. (3) Eastern Cooperative Group (ECOG) performance status score of 0–1. (4) BCLC C stage. (5) Largest tumor lesion diameter ≥5 cm. (6) No prior treatment for HCC. Conversely, participants were excluded if they fulfilled any of the following criteria: (1) ALBI grade 3. (2) Inconsistent administration of HAIC or immunotherapy. (3) Lack of clinical follow‐up. (4) Coexistence of other primary malignancies.

### 2.2 Treatment procedure

All HAIC treatments were performed using digital subtraction angiography by veteran interventional surgeons according to a standard protocol. Under local anesthesia, the modified Seldinger method was performed to puncture the femoral artery and sequentially inserted the 5F vascular sheath. Then, the 5‐Fr Yashiro catheter (Terumo, Tokyo, Japan) was respectively inserted into the celiac trunk and superior mesenteric artery for angiography. After the tumor supply artery was clarified by radiography, the 2.7 F microcatheter (ASAHI, Tokyo, Japan) was coaxially inserted and super‐selected to the tumor blood supply branch artery, the microcatheter head end position was determined by micro‐ductal angiography. After the microcatheter pathway was constructed, a modified FOLFOX6 protocol, which included the administration of oxaliplatin at a dosage of 85 mg/m^2^ via intravenous drip over a period of 2 h on Day 1, calcium folinate at a dosage of 400 mg/m^2^ via intravenous drip over a period of 2 h on Day 1, and fluorouracil at a dosage of 400 mg/m^2^ via intravenous injection on Day 1, followed by a continuous infusion of 2400 mg/m^2^ over 46 h, was delivered through a microcatheter. The combined treatment group continued to receive anti‐PD1 treatment within 2 days after the HAIC. The category and dosage of PD‐1 inhibitors used in the HAIC‐PD1 group were exhibited in **Table S1**. HAIC produce was repeatedly operated before each cycle. HAIC procedure was repeated every 3 weeks until tumor progression. Based on the judgment of adverse events or special causes, chemotherapy drugs (mainly oxaliplatin) and anti‐PD1 drugs can be reduced or discontinued in advance as appropriate. Review of the enhancement CT every 6 weeks. According to mRECIST criteria to assess response to treatment. The follow‐up deadline is December 2022.

### 2.3 Tumor response assessment

The primary endpoints of the study were OS and progression‐free survival (PFS). We compared these endpoints between the HAIC‐PD1 and HAIC groups. OS was defined as the time from the start of treatment until death from any cause or the most recent follow‐up. PFS was defined as the interval from the initiation of tumor treatment to disease progression, as evaluated based on the modified Response Evaluation Criteria in Solid Tumors^22^ (mRECIST), or death from any cause. The efficacy of HAIC was assessed using the mRECIST criteria, which classified responses as complete response (CR), partial response (PR), stable disease (SD), or progressive disease (PD). Two experienced radiologists independently conducted all radiographic evaluations of tumor response. The secondary endpoints included the objective response rate (ORR) which represented the proportion of patients who achieved a tumor response categorized as CR or PR, and the disease control rate (DCR) which was defined as the percentage of patients who achieved a tumor response categorized as CR, PR, or SD. Adverse events (AEs) were assessed according to the Common Terminology Criteria for Adverse Events version 5.0.^23^

### 2.4 Propensity score matching analysis

We employed the 1:1 propensity score matching (PSM) method to mitigate the impact of confounding factors and balance the differences between the groups. The matching tolerance was adjusted to 0.02. The variables considered in the PSM analysis included age, gender, ECOG score, comorbidities, HBV infection, ascites, tumor size, number of tumors, vascular invasion, metastasis, ALBI grade, and AFP level.

### 2.5 Statistical analysis

The analysis for this study was conducted using R software (Rstudio version 4.2.2). Continuous variables following a normal distribution were presented as mean ± standard deviation and analyzed using the Student's *t*‐test. For variables that did not follow a normal distribution, the median was used and analyzed using the Mann–Whitney *U*‐test. Categorical variables were reported as percentages and analyzed using either the chi‐squared test or Fisher's exact test. Kaplan–Meier curves were used to estimate the OS and PFS between the groups in both the overall and matched cohorts, employing the Kaplan–Meier method. The Cox proportional hazards regression model was performed to identify independent risk factors influencing OS and PFS. Factors with a *p* < 0.10 in the univariate analysis were included in the multivariate analysis. A two‐tailed *p* < 0.05 was considered statistically significant.

## 3 RESULTS

### 3.1 Study population

**FIGURE 1:**
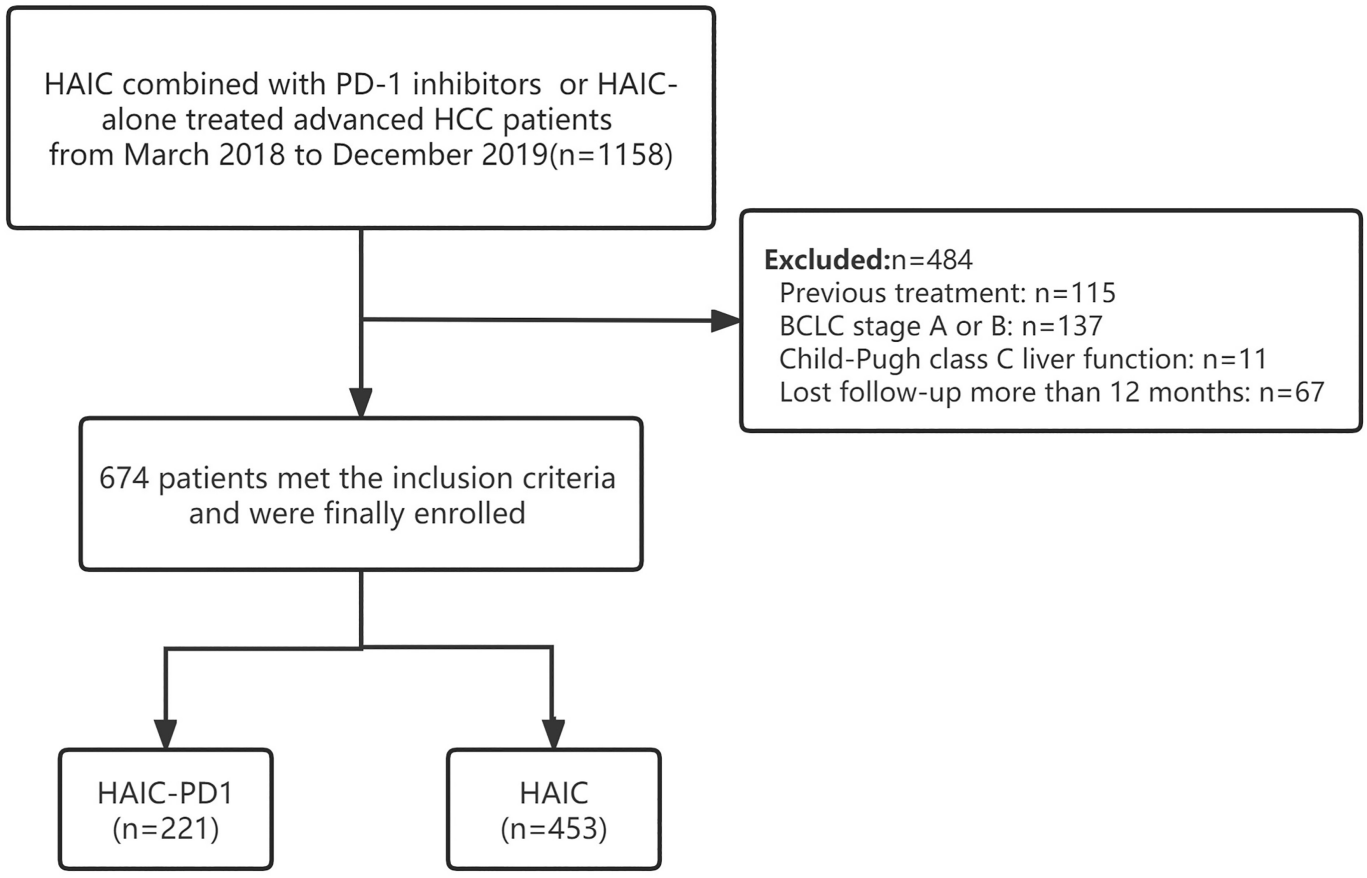
Flowchart of the patient's selection process for this study.

The baseline characteristics of the patients enrolled in this study are presented in Table 1. A total of 674 patients with advanced HCC met the inclusion criteria and were included in the study. Among them, 453 patients received a combination of HAIC and PD1 inhibitors, while the remaining 221 patients received HAIC monotherapy. The patient selection process is illustrated in Figure 1. The majority of the matched cases were male and had a hepatitis B virus infection. Among them, 386 HBV patients (255 [56.3%] patients in the HAIC group vs. 131 [59.3%] patients in the HAIC‐PD1 group, accounting for 59.3%, *p* = 0.462) received continuous antiviral therapy. The average maximum diameter of solid tumors was 11.0 cm, and 265 (59.7%) patients had more than three intrahepatic tumors. Additionally, there were 531 (78.8%) cases with vascular invasion and 354 (52.8%) cases with extrahepatic metastasis. After PSM, a total of 442 patients remained in the study, with 221 patients in the HAIC‐PD1 group and 211 patients in the HAIC group. Before PSM adjustment, a higher percentage of patients in the HAIC alone group had vascular invasion (76.6% vs. 83.3%, *p* = 0.047) and poor liver function reserve (55.2% vs. 46.2%, *p* = 0.028) were observed than in the HAIC‐PD1 group. However, all significant differences between the groups were eliminated after matching.

**TABLE 1:** Baseline characteristics of the study patients before and after PSM.

	Before matching			After matching		
	HAIC‐PD1 (*n* = 221)	HAIC (*n* = 453)	*p* value	HAIC‐PD1 (*n* = 221)	HAIC (*n* = 221)	*p* value
Gender			0.374			0.771
Male	193(87.33%)	406(89.62%)		193(87.33%)	195(88.24%)	
Female	28(12.67%)	47(10.38%)		28(12.67%)	26(11.76%)	
Age			0.100			0.689
≤ 65y	203(91.86%)	404(89.18%)		203(91.86%)	209(94.57%)	
> 65y	18(8.14%)	49(10.82%)		18(8.14%)	12(5.43%)	
ECOG ^a^			0.601			0.478
0	190(85.97%)	396(87.42%)		190(85.97%)	195(88.24%)	
1	31(14.03%)	57(12.58%)		31(14.03%)	26(11.76%)	
Comorbidities			0.644			0.541
Presence	26(11.76%)	59(13.02%)		26(11.76%)	199(90.05%)	
Absence	195(88.26%)	394(86.98%)		195(88.24%)	22(9.95%)	
HBV			0.609			0.856
Presence	204(92.31%)	423(93.38%)		204(92.31%)	205(92.76%)	
Absence	17(7.69%)	30(6.62%)		17(7.69%)	16(7.24%)	
Ascites			0.763			1.000
Presence	36(16.29%)	78(17.22%)		36(16.29%)	36(16.29%)	
Absence	185(83.71%)	375(82.78%)		185(83.71%)	185(83.71%)	
AFP			0.977			1.000
≤ 400 ng/L	91(41.18%)	186(41.06%)		91(41.18%)	91(41.18%)	
> 400 ng/L	130(58.82%)	267(58.94%)		130(58.82%)	130(58.82%)	
ALBI grade			0.028			0.859
1	119(53.85%)	203(44.81%)		119(53.85%)	121(54.75%)	
2	102(46.15%)	250(55.19%)		102(46.15%)	100(45.25%)	
Tumor size			0.083			0.902
≤ 7 cm	41(18.55%)	111(24.50%)		41(18.55%)	40(18.10%)	
> 7 cm	180(81.45%)	342(75.50%)		180(81.45%)	181(81.90%)	
Tumor number			0.717			0.923
1–3	88(39.82%)	187(41.28%)		88(39.82%)	87(39.37%)	
> 3	133(60.18%)	266(58.72%)		133(60.18%)	134(60.63%)	
MVI			0.047			0.707
Presence	184(83.26%)	347(76.60%)		184(83.26%)	181(81.90%)	
Absence	37(16.74%)	106(23.40%)		37(16.74%)	40(18.10%)	
Metastasis			0.776			0.340
Presence	115(52.04%)	241(53.20%)		115(52.04%)	125(56.56%)	
Absence	106(47.96%)	212(46.80%)		106(47.96%)	96(43.44%)	

*Note*: *p* < 0.05 indicated a significant difference.

Abbreviations: AFP, α‐fetoprotein; ALBI, albumin‐bilirubin; ECOG, Eastern Cooperative Oncology Group score; HAIC, hepatic arterial infusion chemotherapy; HAIC‐PD1, hepatic arterial infusion chemotherapy combined with PD‐1 inhibitors. HBV, hepatitis B virus; MVI, microvascular invasion; PSM, propensity score matching.

### 3.2 Long‐term survival outcomes

**FIGURE 2:**
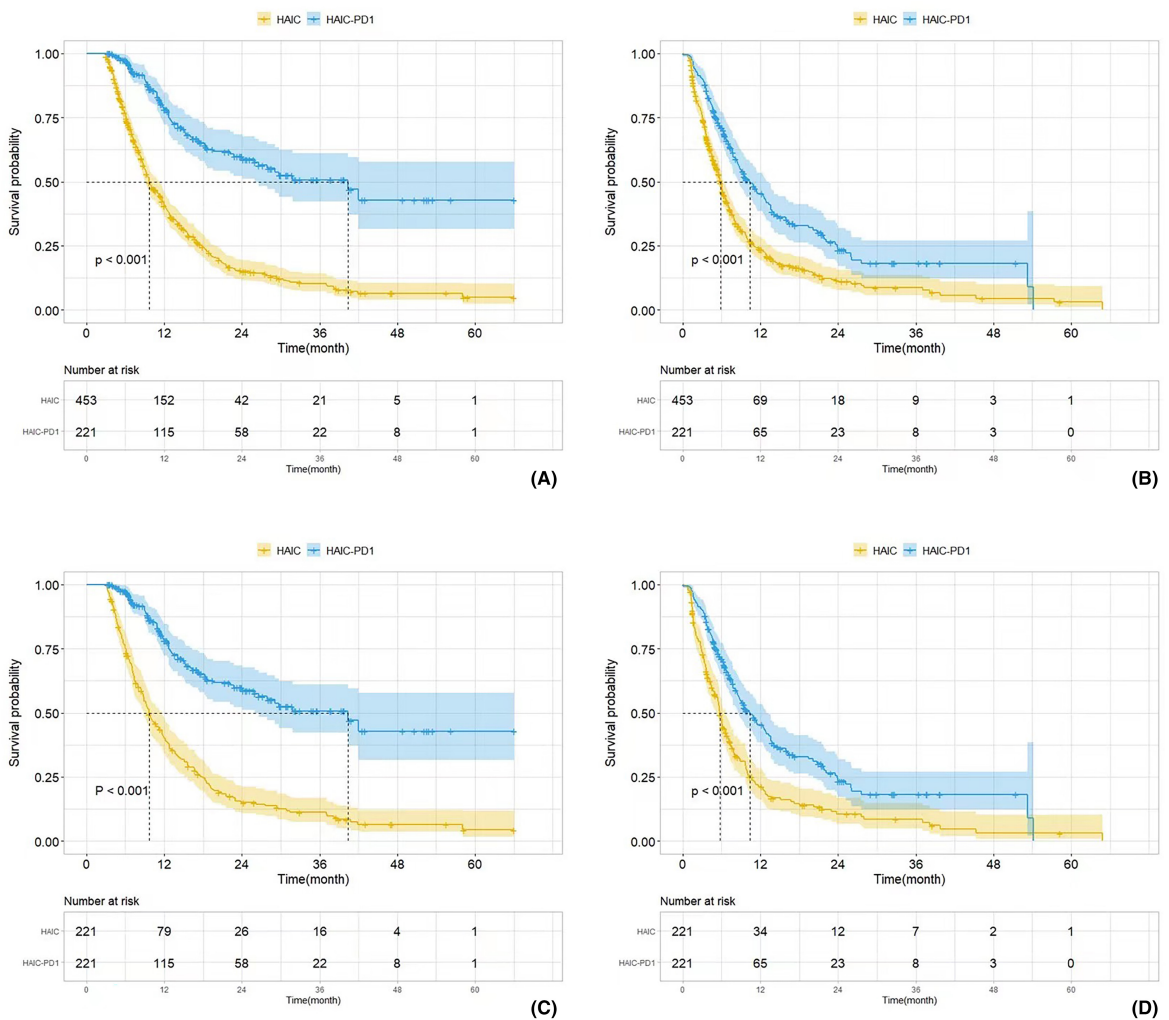
The Kaplan–Meier survival curves by Log‐rank test for the HAIC‐PD1 group and the HAIC group with or without propensity score matching (PSM) adjustment. (A) The Kaplan–Meier curves comparing the overall survival between the HAIC‐PD1 group and the HAIC group, without adjusting for overall survival; (B) The Kaplan–Meier curves comparing the overall survival between the HAIC‐PD1 group and the HAIC group, without adjusting for progression‐free survival (PFS); (C). Comparison of PSM‐adjusted overall survival between the HAIC‐PD1 group and HAIC groups; (D) Comparison of PSM‐adjusted PFS between the HAIC‐PD1 group and HAIC groups.

The entire and PSM‐adjusted OS‐ and PFS‐related K–M curves are shown in Figure 2. The median follow‐up duration was 37.2 months. In the HAIC‐PD1 group, the median overall survival (mOS) was 40.4 months (HR: 4.17, 95% CI: 3.22–5.38), whereas in the HAIC group it was 9.7 months (HR: 0.24, 95% CI: 0.19–0.31). The median progression‐free survival (mPFS) in the HAIC‐PD1 group was 22.10 months (HR: 3.88, 95% CI: 2.95–5.10) compared to 5.70 months (HR: 0.26, 95% CI: 0.20–0.34) in the HAIC group. Both mOS and mPFS showed statistically significant differences between the two groups. One‐, 2‐, and 3‐year OS rates after treatment were 52.0%, 26.2%, and 10.0% in the HAIC‐PD1 group, and 33.6%, 9.3%, and 4.6% in the HAIC group (*p* < 0.001). One‐, 2‐, and 3‐year PFS rates after treatment were 29.4%, 10.4%, and 3.6% in the HAIC‐PD1 group, and 15.2%, 4.0%, and 2.0% in the HAIC group (*p* < 0.001). Among the 221 PSM‐matched pairs, the HAIC‐PD1 group demonstrated a median OS of 40.4 months (HR: 4.18, 95% CI: 3.18–5.50) and a median PFS of 22.1 months (HR: 3.90, 95% CI: 2.97–5.14), compared to 22.1 months (HR: 3.90, 95% CI: 2.97–5.14) and 9.7 months (HR: 0.24, 95% CI: 0.18–0.31) in the unmatched group. The matched groups also exhibited statistically significant differences in long‐term OS and PFS rates. One‐, 2‐, and 3‐year OS rates after treatment were 52.0%, 26.2%, and 10.0% in the HAIC‐PD1 group, and 35.8%, 11.8%, and 7.2% in the HAIC group (*p* < 0.001). One‐, 2‐, and 3‐year PFS rates after treatment were 29.4%, 10.4%, and 3.9% in the HAIC‐PD1 group, and 15.4%, 5.4%, and 3.2% in the HAIC group (*p* < 0.001).

### 3.3 Univariate and multivariate analysis

Univariate and multivariate analyses were conducted to identify predictors of OS and PFS, and the findings are presented in Table 2. In the univariate analysis, significant covariates associated with OS included alpha‐fetoprotein (AFP), the presence of metastasis, and therapeutic schedule. Similarly, HBV infection, AFP, metastasis, and therapeutic schedule were found to be correlated with PFS. Furthermore, the multivariable Cox regression analysis revealed that AFP, metastasis, and therapeutic schedule were significant prognostic factors for OS. Additionally, HBV infection, metastasis, and therapeutic schedule were identified as significant prognostic factors for PFS.

**TABLE 2:** Univariate and multivariate analyses of predictors of survival after treatment.

Factors	Overall survival				Progression‐free survival			
	Univariate analysis *p* value	Multivariate analysis			Univariate analysis *p* value	Multivariate analysis		
		HR	95%CI	*p* value		HR	95%CI	*p* value
Gender	0.554	‐	‐	‐	0.530	‐	‐	‐
Male								
Female								
Age	0.268	‐	‐	‐	0.445	‐	‐	‐
≤ 65y								
> 65y								
ECOG	0.904	‐	‐	‐	0.315	‐	‐	‐
0								
1								
Comorbidities	0.312	‐	‐	‐	0.354	‐	‐	‐
Presence								
Absence								
HBV	0.093	‐	‐	‐	0.033	2.129	1.281,3.539	**0.004**
Presence								
Absence								
Tumor size	0.194	‐	‐	‐	0.110	‐	‐	‐
≤7 cm								
>7 cm								
Ascites	0.962	‐	‐	‐	0.838	‐	‐	‐
Presence								
Absence								
Tumor number	0.120	‐	‐	‐	0.056	‐	‐	‐
1–3								
> 3								
MVI	0.447	‐	‐	‐	0.644	‐	‐	‐
Presence								
Absence								
Metastasis	0.045	1.357	1.057,1.744	**0.016**	0.007	1.481	1.152,1.904	**0.002**
Presence								
Absence								
ALBI grade	0.546	‐	‐	‐	0.910	‐	‐	‐
1								
2								
AFP	0.045	1.360	1.055,1.752	**0.017**	0.027	1.403	1.095,1.823	**0.010**
≤400 ng/mL								
>400 ng/mL								
Treatment method	<0.001	0.268	0.203,0.353	**<0.001**	<0.001	0.258	0.195,0.340	**<0.001**

*Note*: Bold indicates statistical significance level at *p* < 0.05.

Abbreviations: AFP, α‐fetoprotein; ALBI, albumin‐bilirubin; CI, confidence interval; ECOG, Eastern Cooperative Oncology Group score; HBV, hepatitis B virus; HR, hazard ratios; MVI, Microvascular invasion.

### 3.4 Radiological response

The best treatment responses before and after PSM are shown in Table 3. Based on mRECIST criteria, in the HAIC‐PD1 group, tumor responses consist of 0.9% of CR, 23.1% of PR, 60.6% of patients with SD, and 8.1% of PD. In the HAIC alone group, including 0.4% of CR, 21.6% of PR, 60.7% of SD, and 17.2% of patients with PD. Without adjustment, the HAIC‐PD1 was associated with a higher ORR (42.5% vs. 22.1%, *p* < 0.001) and DCR (91.9% vs. 82.8%, *p* = 0.002) compared to HAIC‐alone. After propensity score weighting, HAIC remains significantly relevant to a higher rate of tumor response ORR (42.5% vs. 24.0%, *p* < 0.001) and DCR (91.9 vs. 83.3%, *p* = 0.018). In addition, treatment schedules follow‐up after the PD of HAIC‐PD1 or HAIC therapy are shown in **Table S2**.

**TABLE 3:** Best tumor response before and after PSM.

	Before PSM			After PSM		
	HAIC‐PD1 (*n* = 221)	HAIC (*n* = 453)	*p* value	HAIC‐PD1 (*n* = 221)	HAIC (*n* = 221)	*p* value
Best Response			<0.001			<0.001
CR	1(0.5%)	1(0.2%)		1(0.5%)	1(0.5%)	
PR	93(42.1%)	93(20.5%)		93(42.1%)	51(23.1%)	
SD	109(49.3%)	275(60.7%)		109(49.3%)	134(60.6%)	
PD	18(8.1%)	79(17.4%)		18(8.1%)	35(15.8%)	
ORR	42.5%(94/221)	20.8%(94/453)	<0.001	42.5%(94/221)	23.5%(52/221)	<0.001
DCR	91.9%(203/221)	81.5%(369/453)	0.001	91.9%(203/221)	84.2%(186/221)	0.013

Abbreviations: CR, complete response; DCR, disease control rate; HAIC, hepatic arterial infusion chemotherapy; HAIC‐PD1; hepatic arterial infusion chemotherapy combined with programmed cell death‐1 inhibitors; ORR, objective response rate; PD, progressive disease; PR, partial response; SD, stable disease.

### 3.5 Subgroup analysis

**FIGURE 3:**
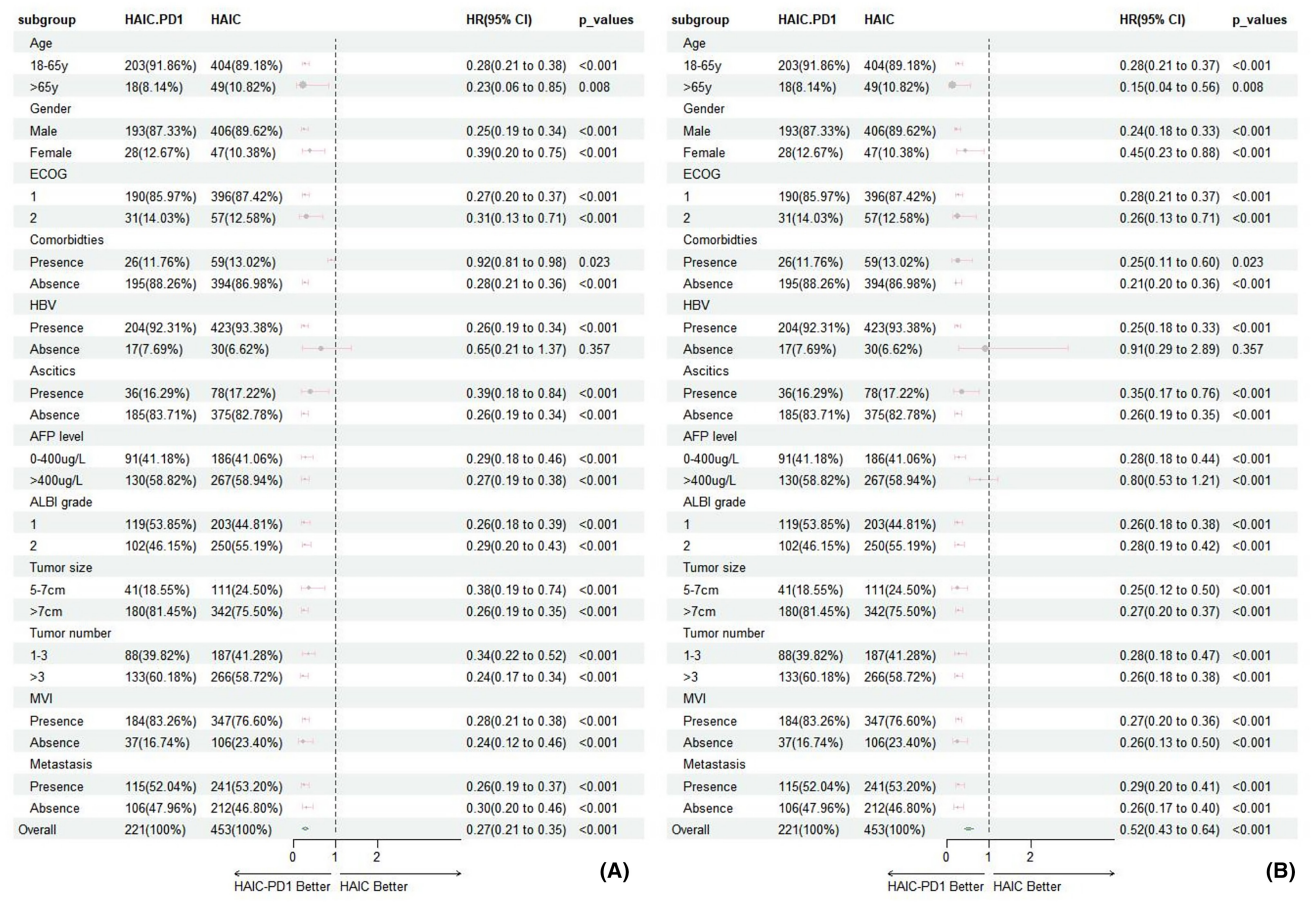
Forestplot based on overall survival (A) and progression‐free survival (B) of each subgroup.

Forest plots (Figure 3) were plotted to illustrate the comparison between subgroups. When analyzing the matched cohort, it was observed that, with the exception of the subgroup without HBV infection, the combination of HAIC and PD1 exhibited enhanced overall survival benefits and more effective tumor progression control in all other subgroups in comparison to the HAIC group. This finding implies that PD1 inhibitors may have even more promising efficacy in the treatment of advanced HCC in patients with HBV infection.

### 3.6 Safety

The occurrence of adverse reactions between the HAIC‐PD1 combination group and the HAIC monotherapy group revealed no significant difference (Table 4). It is worth noting that all adverse reactions were manageable, and symptomatic treatment led to significant relief. Importantly, no treatment‐related deaths attributed to drug toxicity were reported during the treatment period. The most frequently observed adverse events in the HAIC‐PD1 group included elevated transaminase levels, abdominal pain, and neutrophil reduction. Conversely, the HAIC group exhibited elevated transaminase levels, abdominal pain, and fatigue as the most common adverse events.

**TABLE 4:** Treatment‐related adverse events.

Adverse events	Any grade			Grade 3/4		
	HAIC‐PD1 (*n* = 221)	HAIC (*n* = 453)	*p* value	HAIC‐PD1 (*n* = 221)	HAIC (*n* = 453)	*p* value
Treatment‐related AEs, *n* (%)				9(4.1)	9(2.0)	186
Hypertension	24(10.9)	57(12.6)	0.603	1(0.5)	0(0)	0.714
Fatigue	55(24.9)	92(20.3)	0.221	0(0)	0(0)	1.000
Fever	43(19.5)	67(14.8)	0.153	3 (1.4)	0 (0)	0.062
Abdominal pain	57(25.8)	96 (21.1)	0.215	0 (0)	1 (0.2)	0.814
Gingival bleeding	22(10.0)	36(7.9)	0.709	2(1)	5(1.1)	1.000
Neurologic toxicity	12(5.4)	20(4.4)	0.697	0(0)	0(0)	1.000
Hypothyroidism	5(2.3)	2(0.4)	0.074	0(0)	0(0)	1.000
Hyperthyroidism	3(1.4)	3(0.7)	0.642	0(0)	0(0)	1.000
Pneumonitis	4(1.8)	9(2.0)	1.000	0(0)	0(0)	1.000
**Respiratory**						
Cough	35(15.3)	73(16.1)	1.000	0(0)	0(0)	1.000
dyspnea	4(1.8)	5(1.1)	0.509	0(0)	0(0)	1.000
**Dermatologic events**						
Rash	10 (4.5)	17 (3.8)	0.787	0 (0)	0 (0)	1.000
Hand‐foot syndrome **Gastrointestinal events**	34(15.4)	47(10.4)	0.080			
Diarrhea	25 (11.3)	63 (13.9)	0.414	0 (0)	1 (0.2)	1.000
Vomiting	22 (10.0)	47 (10.4)	0.973	2(1)	2(0.4)	0.840
Nausea	17 (7.7)	44 (9.7)	0.474	0 (0)	0 (0)	1.000
Inappetence	6 (2.7)	8 (0)	0.601	1 (0.5)	0(0)	
**Laboratory‐related AEs, *n* (%)**				6(2.7)	5(1.1)	0.220
**Hematotoxicity**						
Anemia	22 (10.0)	51(11.3)	0.124	1(0.5)	0(0)	
Leukopenia	38 (17.2)	59(13.0)	0.183	0(0)	0(0)	1.000
Neutropenia	47 (21.3)	88 (19.4)	0.647	2(1)	1(0.2)	
Thrombocytopenia	27 (12.2)	59 (13.0)		1(0.5)	1(0.2)	
**Hepatotoxicity**						
Elevated ALT	81 (36.7)	133 (29.4)	0.864	1(0.5)	1(0)	
Elevated AST	74 (33.5)	141 (31.1)	0.597	1(0.5)	2(0.4)	
Hypoalbuminemia	2 (0.9)	0 (0)	0.203	0(0)	0(0)	1.000
Hyperbilirubinemia	1 (0.5)	0 (0)	0.714	0(0)	0(0)	1.000
**Nephrotoxicity**						
Elevated creatinine	6 (2.7)	10 (2.2)	0.891	0(0)	0(0)	1.000
Proteinuria	3 (1.4)	2 (0.4)	0.411	0(0)	0(0)	1.000

Abbreviation: AEs, adverse events.

## 4 DISCUSSION

From the perspective of genetics and epigenetics, HCC is a malignancy characterized by marked intratumoral heterogeneity, the difficulty of capturing which to a certain degree determines the sensitivity and plasticity of tumor cells to different treatment modalities. In comparison, treatment strategies in compliance with tumor burden and liver function appear to be currently suboptimal. In the absence of precise therapies based on intratumoral heterogeneity profiling, immunotherapy‐based combination therapy represents a promising direction in augmenting tumor response.^24^ As one of the few available local treatment options, HAIC is also of great significance in managing the progression and prolonging survival of unresectable HCC patients. In our retrospective study, we utilized PSM to eliminate group differences, enrolled a larger number of cases across multiple centers, and conducted long‐term follow‐ups. Eventually, irrespective of whether in the entire cohort or in the PSM cohort, consistent conclusions for discrepant OS and PFS between groups were acquired. In the matched cohort of this study, a notable mOS of 40.4 months and advantageous ORR and DCR of 42.5% and 91.9% were obtained in the HAIC‐PD1 group, respectively.

In the era of immunotherapy, anti‐PD1 treatment plays a crucial role in the treatment of solid tumors. However, monotherapy with immune checkpoint inhibitors has shown low response rates for tumor regression. For example, the ORR of nivolumab, pembrolizumab, and cemiplimab are 20%, 17%, and 14.7%, respectively, with limited OS benefits.^15^, ^25^, ^26^ The main reason for this is that blocking the PD1/PD‐L1 axis alone is insufficient to induce effective anti‐tumor activity.^27^ In recent years, significant achievements have been made in combination therapies involving immunotherapy. According to the results reported from the IMbrave150 trial, the combination of atezolizumab and bevacizumab achieved an encouraging median survival time of 19.2 months, significantly surpassing the 13.4 months of the sorafenib group, particularly for the subset in China reaching a breakthrough 24.0 months.^28^ Another randomized trial reported a median OS of 18.7 months and a response rate of 24.0% based on the dual immunotherapy regimen of tremelimumab combined with durvalumab, which outperformed monotherapy immunotherapy.^29^ Subsequently, atezolizumab plus bevacizumab and tremelimumab plus durvalumab have been recommended as first‐line treatments. Additionally, the combination of anti‐PD1 treatment with local therapies has shown potential. XIn et al. reported that in the treatment of advanced HCC, the combination of HAIC with atezolizumab and bevacizumab achieved a remarkable ORR of 67.3% based on the mRECIST criteria and 44.2% based on the RECIST1.1 criteria.^30^ A retrospective study conducted by Mei et al. showed that adding anti‐PD1 treatment to HAIC combined with lenvatinib extended the mOS of patients with advanced HCC by 7 months.^31^ Moreover, significant survival benefits from the combination of anti‐PD1 treatment with TACE have been confirmed by multiple studies.^32^, ^33^

Currently, combined immunotherapy of atezolizumab plus bevacizumab or durvalumab plus tremelimumab was significantly superior to standard sorafenib in prognosis according to these two clinical trials. Indeed, TKIs sequential therapy has been more effective in improving prognosis than single TKIs. However, Kudo pointed out that the favorable prognosis achieved with TKIs sequential therapy can only be attained in the presence of long‐term sustained better liver function, and even slight impairment of liver function can also severely impact treatment efficacy.^34^, ^35^ Moreover, the use of TKIs in systemic therapy is an important factor contributing to elevated transaminase levels, which seem contradictory. In contrast, anti‐PD1 therapy has shown better tolerability with regards to liver function in patients. In this study, a considerable proportion of patients with ALBI Grade 2 were included, and satisfactory outcomes were ultimately achieved in terms of OS, PFS and ORR. On this issue, the combination of HAIC and anti‐PD1 treatment appears to be equally sensitive in patients with relatively poor liver function, a viewpoint also supported by previous research. On the other hand, patients with BCLC stage C represent a subset with poorer liver function, with most of them having a high burden of intrahepatic tumors and portal vein involvement, leading to the development of portal hypertension and worsening of liver function. Therefore, for the population at high risk of liver function deterioration in BCLC stage C, the combination of HAIC and anti‐PD1 treatment appears to be a more effective choice.

The results suggest a benefit correlated with the combined application of HAIC and PD‐1 blockades, which may be attributable to the synergistic effect between these two treatment regimens. Oxaliplatin, as the main chemotherapeutic drug in HAIC, through binding to the antigen on the cell membrane, induces apoptotic cells to release ATP and produces a strong immunogenic cell death (ICD) effect to enhance immune activation in tumor cell microcirculation, thereby synergizing with immune check inhibitors to enhance the curative effect.^36^, ^37^, ^38^, ^39^ Moreover, the administration of chemotherapy augments the antigenicity of tumors by eliciting ICD, thereby diminishing “off‐target” immunosuppression within the tumor microenvironment. This, in turn, intensifies the immune response, regulates tumor progression and metastasis, and closely correlates with the notable response rate observed in the HAIC‐PD1 group in this study. These findings underscore the profound impact of PD‐1 inhibitors on the systemic immune microenvironment, as well as the synergistic interactions arising from the concurrent utilization of catheter‐based chemotherapy and immunotherapy, which exert a pivotal role in restraining disease progression.^40^

In relation to subgroup analyses, notably, the integration of PD1 inhibitors with HAIC as compared to HAIC monotherapy exhibited significantly improved OS and PFS outcomes, irrespective of tumor size, tumor number, or the presence of vascular invasion. These findings suggest that HAIC‐PD1 may offer a superior therapeutic approach for advanced HCC, regardless of the extent of tumor burden. Furthermore, the amalgamation of HAIC and PD1 demonstrates not only a therapeutic impact on intrahepatic lesions but also confers a survival benefit on patients with advanced HCC characterized by extrahepatic metastases. Besides, the HAIC‐PD1 regime failed to attain superior survival outcomes compared to the HAIC monotherapy group only within the non‐hepatitis B‐related HCC subgroups. Possible underlying factors are as follows, on the one hand, prior researches have demonstrated that upregulation of regulatory factors such as PD‐1, CTLA‐4, and TIM‐3 serves as a significant mechanism of immune dysfunction induced by chronic hepatitis B virus infection.^41^ Blockade of immune checkpoints may endeavor to enhance immune control through addressing these molecular pathways. On the other hand, checkpoint inhibitors and other immune‐modulating agents can help to curtail T cell exhaustion and even promote their reinvigoration.^42^ Given the existing research, the risk of HBV reactivation remains rarely defined in cancer patients receiving immune checkpoint inhibitors, further in‐depth research is needed to clarify the underlying mechanisms involved.

In the present study, the utilization of multivariate analysis revealed that AFP and metastasis emerged as common and statistically significant risk factors linked to OS and PFS. Furthermore, the examination of tumor tissue morphology demonstrated a correlation between heightened levels of HCC tumor markers and the extent of lesion invasion, encompassing vascular invasion and metastasis.^43^ Hitherto, several Phase III studies acknowledged persistent high AFP levels as a relevant factor for unsatisfactory survival and lesion progression.^44^, ^45^ As well, this study provides evidence that HAIC‐PD1 is an independent risk factor affecting OS and PFS in advanced‐staged HCC patients. These findings support the positive effect of HAIC‐PD1 in late‐stage HCC and suggest that it may represent a promising therapeutic strategy for improving clinical outcomes. In terms of drug‐related toxicity, the combined application of HAIC and PD‐1 inhibitors is safe and well‐tolerated. The HAIC‐PD1 group did not demonstrate a significantly higher incidence of adverse events. The occurrence of Grade 3–4 adverse events was similar between the groups. Most adverse events were relieved with symptomatic treatment and did not worsen further.

In view of the current limitations of the administration of HAIC and targeted‐immunotherapy, the synergistic effect of the combination of multiple treatment options can help remedy this deficiency. A number of current studies on combination therapy have been carried out one after another, aiming to explore individualized treatment options that can provide more survival benefits for different patients with tolerable side effects. The conclusions of similar studies related to HAIC in different centers seem to be contradictory, which may also be attributed to different HAIC or different dosing regimens of systemic therapy. Therefore, in the follow‐up research, efforts should be made to standardize the therapeutic schedule and eliminate the differences caused by different treatment methods.

There is necessity to acknowledge that the present study has certain limitations due to its retrospective nature, which inherently introduces unavoidable selection bias. While we employed PSM to minimize between‐group differences, it is possible that endogenous differences may still exist. Thus, there is a need for additional prospective randomized controlled trials to enhance the level of evidence and further validate our findings. Furthermore, it is crucial to highlight that our study was conducted in the specific milieu of China, a nation characterized by a substantial prevalence of hepatitis B virus. This circumstance warrants careful consideration, as it has the potential to impose restrictions on the extent to which our findings can be extrapolated to diverse populations. In addition, it is worth considering that different anti‐PD1 drugs may yield varying research outcomes, thus necessitating a more rigorous experimental design to enhance methodological rigor. Lastly, this was a multicenter study, the HAIC process may have variations among different centers, which may affect the outcomes.

In conclusion, this study provides suggestion that the concomitant use of HAIC and PD‐1 inhibitors offers advantageous survival benefits for patients with advanced HCC, while also being well‐tolerated. Importantly, the combined application of HAIC and anti‐PD‐1 therapy demonstrates a more substantial survival advantage in patients with late‐stage HCC compared to HAIC monotherapy.

## AUTHOR CONTRIBUTIONS

**Yangyang Li:** Data curation (lead); methodology (lead); resources (lead); software (lead); supervision (lead); writing – original draft (lead). **Wendao Liu:** Conceptualization (equal); methodology (equal); resources (equal); writing – original draft (equal). **Junwei Chen:** Conceptualization (equal); methodology (equal); supervision (equal). **Yongxin Chen:** Methodology (equal); writing – original draft (equal). **Jiandong Guo:** Formal analysis (equal); resources (equal); software (equal). **Huajin Pang:** Data curation (equal); resources (equal); validation (equal). **Wentao Zhang:** Methodology (equal); resources (equal); visualization (equal). **Chao An:** Conceptualization (equal); project administration (equal); writing – review and editing (equal). **Chengzhi Li:** Conceptualization (lead); methodology (lead); project administration (lead); writing – review and editing (lead).

## CONFLICT OF INTEREST STATEMENT

The authors of this manuscript assert non‐affiliation or financial association with companies whose products or services are pertinent to the subject matter discussed in the article.

## CONSENT STATEMENT

Patients included in this study were required to obtain written informed consent from each participating center. The use of clinical data from patients included in this study was approved by respective center committees.

## Supporting information


Table S1.
Table S2.

## Data Availability

The data utilized in this study is available upon request. Readers interested in accessing the data can contact [Chengzhi Li, lichengzhi@jnu.edu.cn] for further details.
