# Supplementary material for: Efficiency and safety of hepatic arterial infusion chemotherapy (HAIC) combined with anti‐PD1 therapy versus HAIC monotherapy for advanced hepatocellular carcinoma: A multicenter propensity score matching analysis
Source: Cancer Med. 2024 Jan 9;13(1):e6836. doi: 10.1002/cam4.6836 (PMC10807563; doi:10.1002/cam4.6836)
Supplement: Supplementary file 1 — Table S1. Table S2. [file CAM4-13-e6836-s001.docx]

Supplement Tables

**Table S1.** Category and dosage of PD-1* inhibitors used in the HAIC-PD1 group.

| **Category** | **Dose (mg)** | **HAIC-PD1*(n=221)** |
| --- | --- | --- |
| **Nivolumab**  (Bristol-Myers Squibb  Holdings Pharma, Ltd.Liability Company) | 240 | 13(5.9) |
| **Keytruda** | 200 | 50(22.6) |
| (Carlow, Merck Sharp & Dohme Ireland Corp) |  |  |
| **Toripalimab** | 240 | 68(30.8) |
| (Suzhou, hezhong pharmaceutical Co.Ltd) |  |  |
| **Sintilimab** | 200 | 58(26.2) |
| (Suzhou, xinda pharmaceutical Co.Ltd)) |  |  |
| **Camrelizumab** | 200 | 32(14.5) |
| (Jiangsu Hengrui Medicine Co.,Ltd.) |  |  |

Data represented by n(%)

*Abbreviations: PD-1: programmed cell death protein-1; HAIC-PD1: hepatic infusion chemotherapy plus programmed cell-death-protein-1 inhibitors

**Table S2.** Treatment schedules follow-up after the progressive disease of HAIC-PD1 or HAIC therapy

| **Treatment schedules** | | **HAIC-PD1(n=18)** | **HAIC (n=79)** |
| --- | --- | --- | --- |
| TACE | 9(50.0) | 44(55.7) |  |
| Radiotherapy | 2(11.1) | 13(16.5) |  |
| Ablation | 1(5.6) | 7(8.9) |  |
| TKIs | 16(88.9) | 51(64.6) |  |

Data represented by n(%)

*Abbreviations: HAIC: hepatic infusion chemotherapy; HAIC-PD1: hepatic infusion chemotherapy plus programmed cell-death-protein-1 inhibitors; TACE: transcatheter arterial chemoembolization; TKIs: tyrosine kinase inhibitors.
